# Improving prevention, recognition and treatment of noma

**DOI:** 10.2471/BLT.19.243485

**Published:** 2020-04-08

**Authors:** Alexandra Caulfield, Tobias Alfvén

**Affiliations:** a Department of Global Public Health, Karolinska Institutet, Tomtebodavägen 18, 17177 Stockholm, Sweden.

Global consultations are ongoing in preparation for the launch of World Health Organization’s (WHO) new roadmap for neglected tropical diseases. These consultations will set out the strategic direction for treatment and prevention of these diseases in the coming decade. We therefore argue that it is timely to assess the global progress on noma, a disease endemic to tropical areas and neglected in terms of detection, prevention, treatment, research and policy.

Noma is caused by a polymicrobial bacterial infection of the gums, which progresses rapidly to necrotizing gangrene of the face and jaw.^1^ This disease predominately affects children younger than six years in sub-Saharan Africa and is strongly associated with poor living conditions.^2^ Available estimates of global incidence and prevalence are several decades old, and are not based on epidemiological surveys. However, in Nigeria, researchers have estimated that in 2010–2018 the national incidence ranged from 4.1 to 17.9 per 100 0000 population.^3^

Although patients with noma can be cured with antibiotics,^1^ mortality remains high; nine out of 10 children who contract noma die from the disease.^2^ Survivors face lifelong stigma and disability, affected by sequelae including salivary incontinence, dysphagia and dysphonia.^2^ Currently, the recommended treatment for acute noma is empirical; that is, the choice of antibiotics do not account for the drug sensitivities of the causative bacteria, and little is known about the possible presence of multidrug resistance microorganisms.^4^

Surgery is an option for those who survive, but it is technically complex, costly and often only partially successful.^1^ Access to both surgery and rehabilitative physiotherapy is inequitable, based on geographical accessibility and affordability. Children must wait for the infection to subside for over a year before surgery is even considered and capacity for performing this type of surgery in noma-endemic countries is limited. Most often, access to surgery and physiotherapy depends on the local activity of nongovernmental organizations.

Noma’s devastating impact in these settings has been recognized for a long time,^5^ yet the disease remains neglected. On a local level, health workers often lack the knowledge and skills for early detection and treatment.^6^ Patient advocacy groups are largely non-existent, since the disease affects children from vulnerable groups. On a national level, the governments of countries where noma is endemic may not acknowledge the disease because of its strong association with poor living conditions. Children who have noma are therefore undetected due to the lack of monitoring and surveillance, and ignored due to the lack of political willingness. In addition, other stakeholders, including international donors, academia, research and development actors, health activists and the media, do not include noma in policy and action plans, undermining progress against the disease. Little impetus to recognize noma exists, whether driven by policy change or by communities and other stakeholders. Indeed, noma is so neglected that it does not currently feature in WHO’s portfolio of neglected tropical diseases, which is under scrutiny this year as the roadmap is being finalized.

Defining inclusion criteria for entry into the portfolio of neglected tropical diseases has been problematic. In January 2016, WHO, for the first time, defined the process through which countries can request the inclusion of a disease in this portfolio.^7^ Diseases must meet specified criteria and advocates must provide a dossier of supporting evidence. Noma seems to meet those criteria; it disproportionally affects populations living in poverty in tropical regions, causes morbidity and mortality, stigma and discrimination, and is amenable to broad control or elimination. Furthermore, noma is relatively neglected by research.

However, making a case for noma is challenging. WHO requires a dossier of supporting evidence, and herein lies the problem. If a disease is so neglected that it receives limited funding and consequently little research is conducted, how can evidence be provided to prove that it is neglected? This dilemma was recently identified as a barrier to global reduction of inequities by the United Nations Development Programme.^8^

Two key issues present a challenge for noma’s inclusion in the portfolio. First, WHO is struggling to define neglected tropical diseases, which is understandably a difficult task. Few would argue against the concept of neglected tropical diseases; this category groups together diseases facing similar challenges and provides impetus and funding for diseases that on their own, might gain very little attention. However, not every disease can be included in WHO’s portfolio. So how is a neglected tropical disease defined? Geographical criteria, numbers affected or lack of awareness about that disease? 

Second, defining conditions that do and do not deserve global policy attention will always be problematic. Those who support efforts to contain for the traditional neglected tropical diseases may be unwilling to allow new additions; funding is limited, and prioritization of one issue might detract from others. Nevertheless, we argue that noma can and should be defined as a neglected tropical disease at the highest level. Noma affects vulnerable populations who lack a political voice to advocate for their cause. Furthermore, the lack of inclusion of noma can be seen to constitute an abuse of United Nations (UN) Human Rights Council Resolution, the right to food,^4^ as the disease can impact an individual’s ability to eat, during and after the acute infection. Perhaps most importantly, when symptoms are identified early, noma is fully treatable.^1^

We argue that noma’s absence from WHO’s portfolio of neglected tropical diseases is not justified. We draw a comparison with the infectious disease mycetoma, which was recently included; we do not see any major differences that can account for the different considerations of these two diseases.

How do we turn the needed change into action? In 2019, WHO’s Strategic and Technical Advisory Group on neglected tropical diseases “[noted] with concern the increasing number of diseases proposed for inclusion in the NTD portfolio that are unaccompanied by funded mandates. Resource mobilization, research and advocacy should be encouraged for any condition proposed for inclusion, especially for those that lack adequate information on epidemiology and burden.”^9^ While noma is not explicitly referenced, this statement is clearly relevant to the case of noma.

We therefore call for funding research into the epidemiology of noma, for capacity-building in endemic countries and for health advocacy on local, national and international levels. Proposed actions are to address the epidemiological gaps in knowledge, such as establishing etiology and targeted antimicrobial therapy, and to promote noma’s inclusion in medical curricula, from which it is often omitted. As no policy change can be driven entirely by top-down or bottom-up approaches, actors at all levels must advocate for the acknowledgement and prioritization of noma in the decade to 2030. 

Noma, as well as other diseases fighting for space in the neglected tropical disease list, are not just neglected diseases, but markers of neglected populations. The UN is tracking countries’ progress towards achieving the sustainable development goals (SDGs). The first of these 17 goals is to end poverty in all its forms by 2030.^10^ An ambitious target perhaps, but of fundamental importance to progress across all other spheres; health, education and socioeconomic development.

Leaving no one behind is the SDGs’ core message, and increasingly important as inequality continues to rise within and between countries;^8^ each year since 1980, income inequality has risen, despite an unprecedented global reduction in absolute poverty and economic growth.^11^ The world’s poorest grew relatively poorer, disparities within countries grew wider, and disparities in health outcomes grew wider.^12^ If leaving no one behind is the goal, then policies that target such neglected populations should be the focus.

We hope to add to the growing advocacy movement for noma. *Médecins Sans Frontières* has released in 2019 a documentary about their work with noma in Nigeria as part of a campaign to increase global awareness and action and to coordinate existing local advocacy movements, such as those in Burkina Faso and Niger.^2^ With increasing awareness and advocacy, noma may soon earn its place within the internationally recognized list of neglected tropical diseases, so that in 2030, we can reflect on the progress made in eliminating this preventable and treatable disease. 
